# Supplementary figures and images for: Group B Streptococci lyse endothelial cells to infect the brain in a zebrafish meningitis model
Source: PLoS Biol. 2025 Jul 3;23(7):e3003236. doi: 10.1371/journal.pbio.3003236 (PMC12244714; doi:10.1371/journal.pbio.3003236)

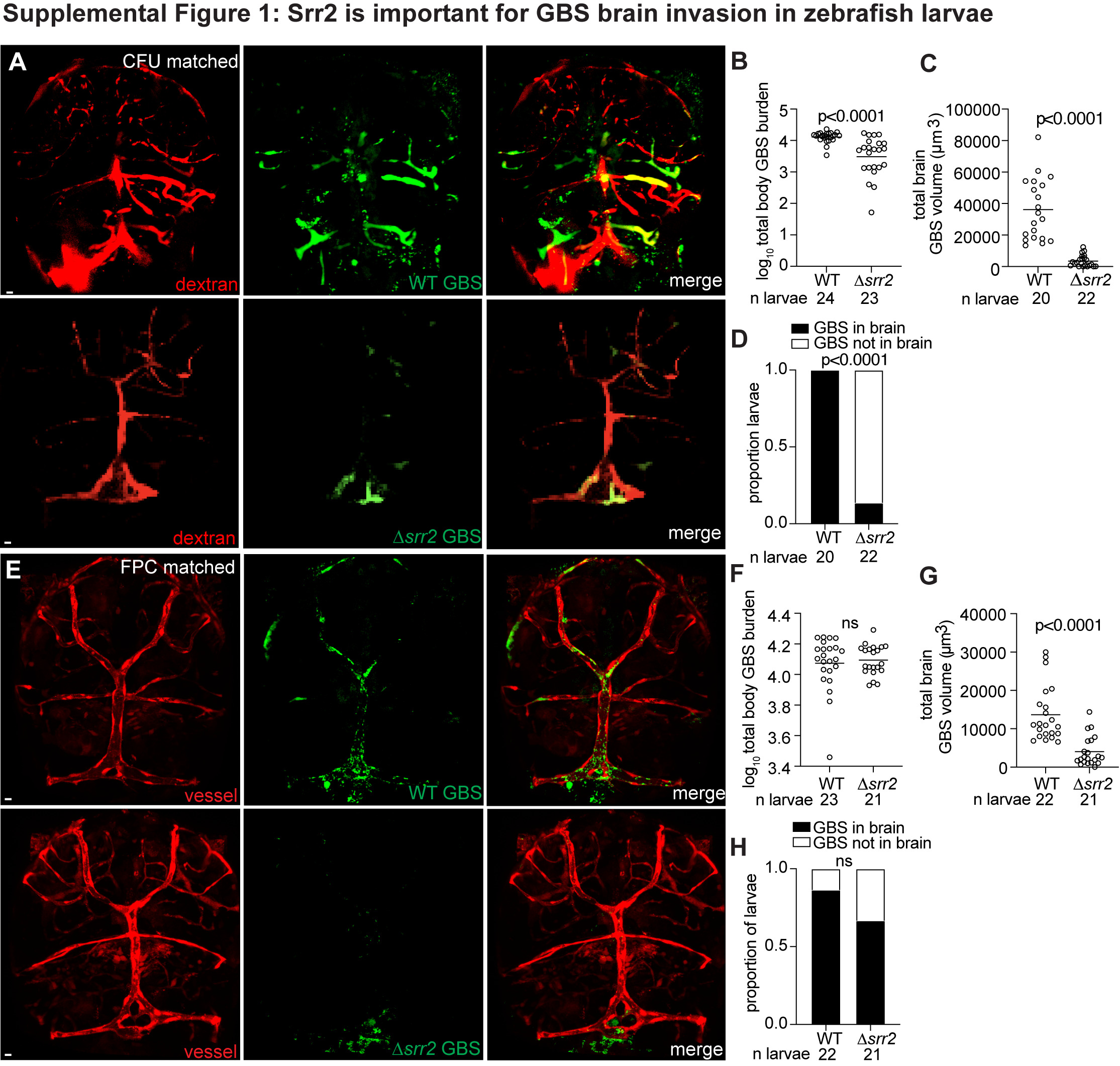

Supplement: S1 Fig — (A) Representative confocal images of brain vasculature labeled by Alexa647 Dextran (pseudocolored red) in 20 hpi larvae infected with approximately 100 CFU wildtype (WT) GBS-GFP (top), or approximately 100 CFU ∆srr2 GBS-GFP (bottom). (B) Wildtype or ∆srr2 burden per larva at 20 hpi quantified by FPC. Horizontal bars, means; Student t test. (C) Quantification of total GBS volume in the brain of wildtype or ∆srr2 GBS infected larvae. Horizontal bars, means; Student t test. (D) Proportion of wildtype or ∆srr2 GBS-infected larvae with GBS in the brain; Fisher’s exact test. (E) Representative confocal images of red fluorescent brain vasculature in 20 hpi larvae infected with approximately 100 CFU wildtype GBS-GFP (top), or approximately 500 CFU ∆srr2 GBS-GFP (bottom). (F) Wildtype or ∆srr2 burden per larva at 20 hpi quantified by FPC. Horizontal bars, means; ns: not significant, Student t test. (G) Quantification of total GBS volume in the brain of wildtype or ∆srr2 GBS infected larvae. Horizontal bars, means; Student t test. (H) Proportion of wildtype or ∆srr2 GBS-infected larvae with GBS in the brain; ns: not significant, Fisher’s exact test. Scale bar, 10 µm throughout. All underlying data in S1 Fig can be found in the supplemental Excel file entitled “S1 Data”. (TIF) [file pbio.3003236.s001.tif]

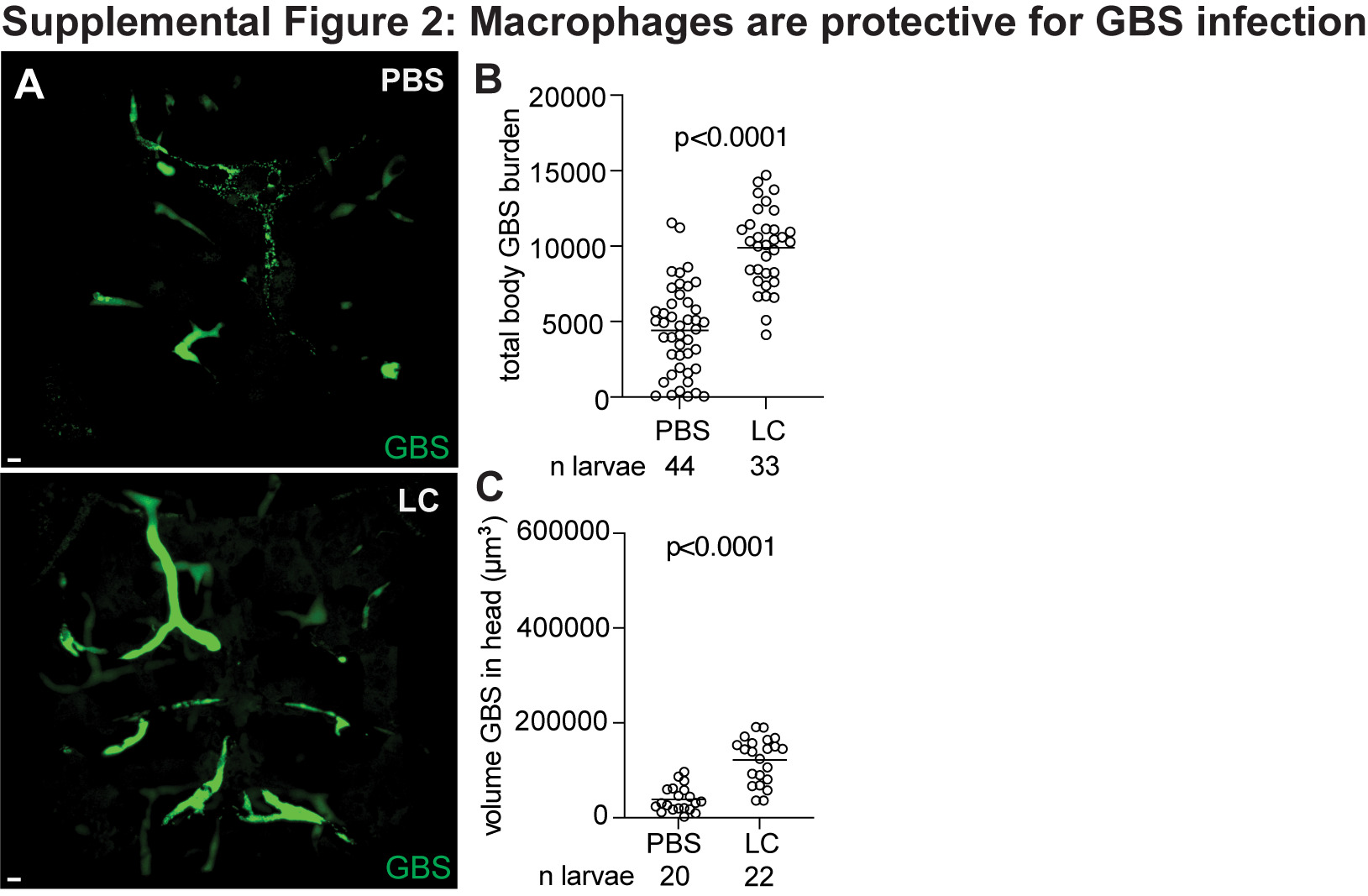

Supplement: S2 Fig — Macrophages are protective for GBS infection. (A) Representative confocal images of brains from a 20 hpi larva infected with approximately 100 CFU GBS-GFP. Larvae were intravenously injected at 2 dpf with PBS (top) or Lipoclodronate (LC) (bottom). Scale bar, 10 µm. (B) GBS burden per larva injected with PBS or LC, quantified by FPC. Horizontal bars, means; Student t test. (C) Quantification of the volume of GBS in the brain of larvae injected with PBS or LC. Horizontal bars, means; Student t test. All underlying data in S2 Fig can be found in the supplemental Excel file entitled “S1 Data”. (TIF) [file pbio.3003236.s002.tif]

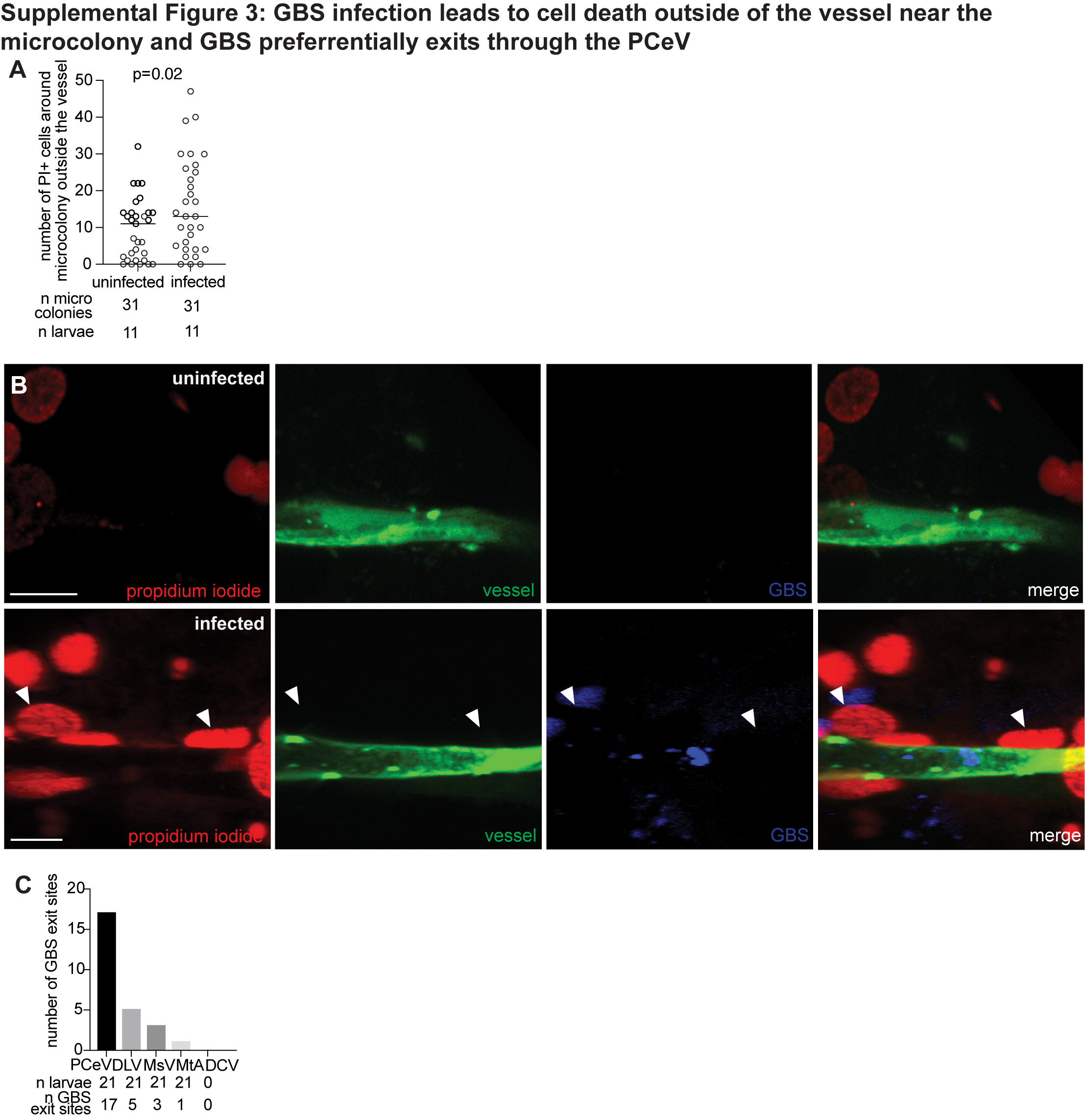

Supplement: S3 Fig — (A) Quantification of propidium iodide (PI) positive staining in non-endothelial cells around the microcolony. Horizontal bars, means; Student t test. (B) Representative confocal images of an uninfected (top) and infected green fluorescent brain blood vessel (bottom) from a 20 hpi larva infected with approximately 100 CFU GBS-BFP and injected with red fluorescent PI just prior to imaging. White arrowheads, cells in the brain labeled with PI. Scale bars, 10 μm. (C) Number of larvae with GBS entering the brain from leptomeningeal vessels, posterior cerebral vein (PceV), dorsal longitudinal vein (DLV), mesencephalic vein (MsV), or metencephalic artery (MtA), or a control vessel, dorsal ciliary vein (DCV). All underlying data in S3 Fig can be found in the supplemental Excel file entitled “S1 Data”. (TIF) [file pbio.3003236.s003.tif]

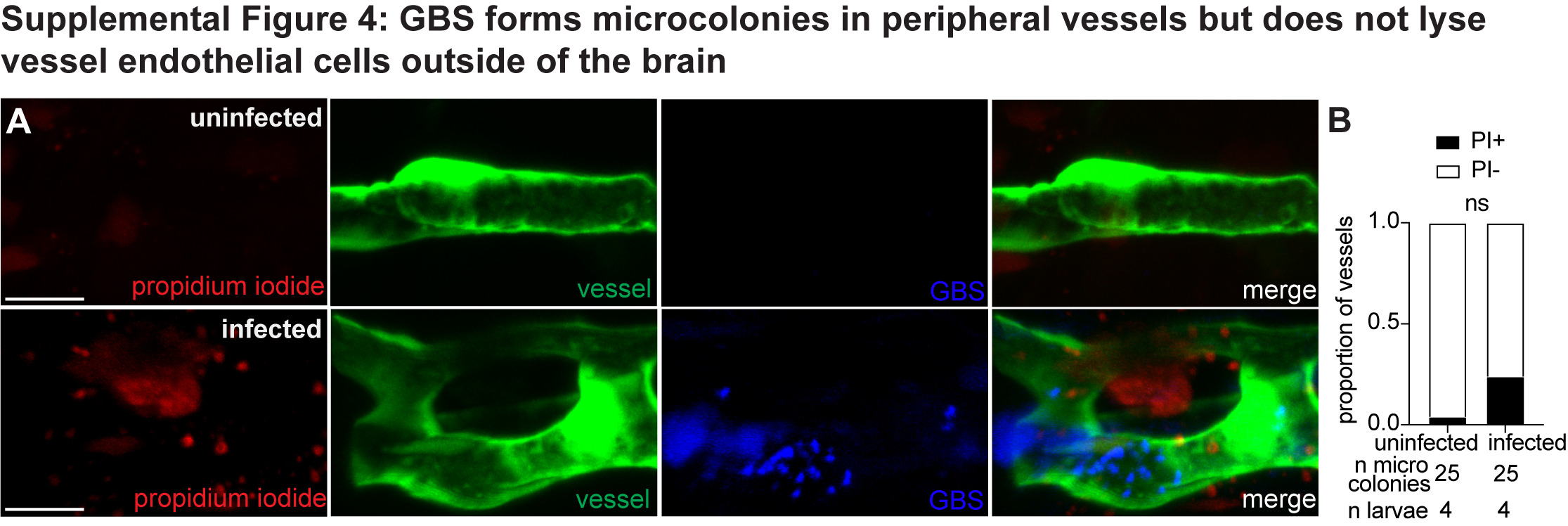

Supplement: S4 Fig — (A) Representative confocal images of an uninfected (top) and infected green fluorescent peripheral (tail) blood vessel (bottom) from a 20 hpi larva infected with approximately 100 CFU blue fluorescent GBS-eBFP and injected intravenously with red fluorescent propidium iodide (PI) just prior to imaging. Scale bars, 10 µm. (B) Proportion of uninfected and GBS-infected vessels with PI-positive nuclei in peripheral vessels; ns: not significant, Fisher’s exact test. All underlying data in S4 Fig can be found in the supplemental Excel file entitled “S1 Data”. (TIF) [file pbio.3003236.s004.tif]

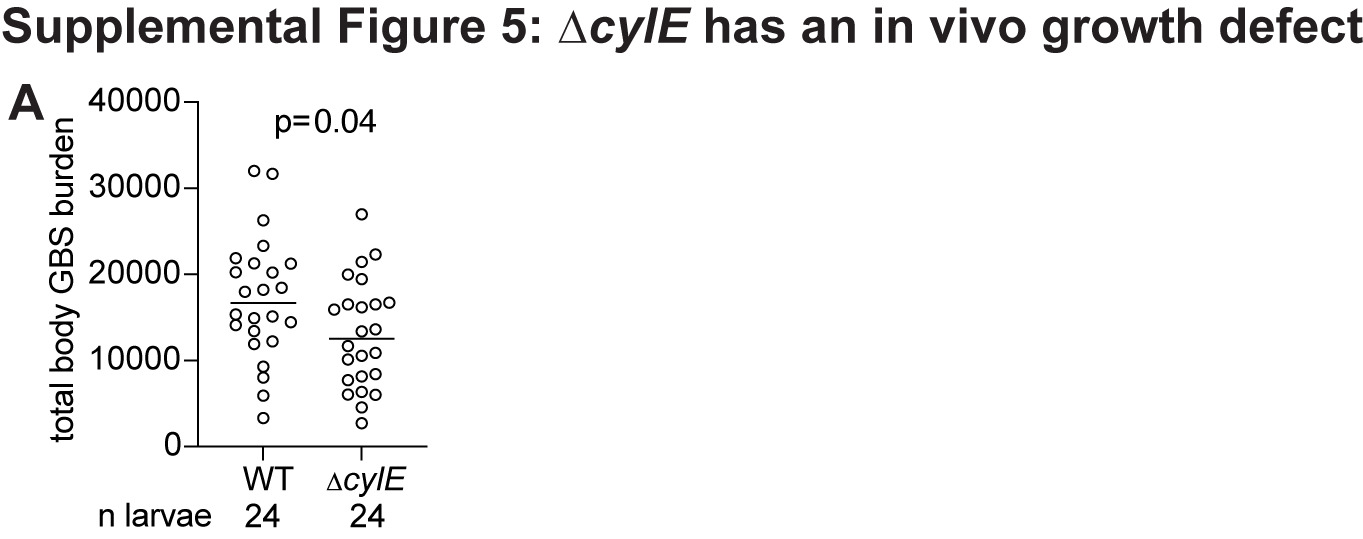

Supplement: S5 Fig — ∆cylE has an in vivo growth defect. (A) Wildtype (WT) or ∆cylE GBS burden per larva at 20 hpi quantified by FPC. Horizontal bars, means, Student t test. All underlying data in S5 Fig can be found in the supplemental Excel file entitled “S1 Data”. (TIF) [file pbio.3003236.s005.tif]

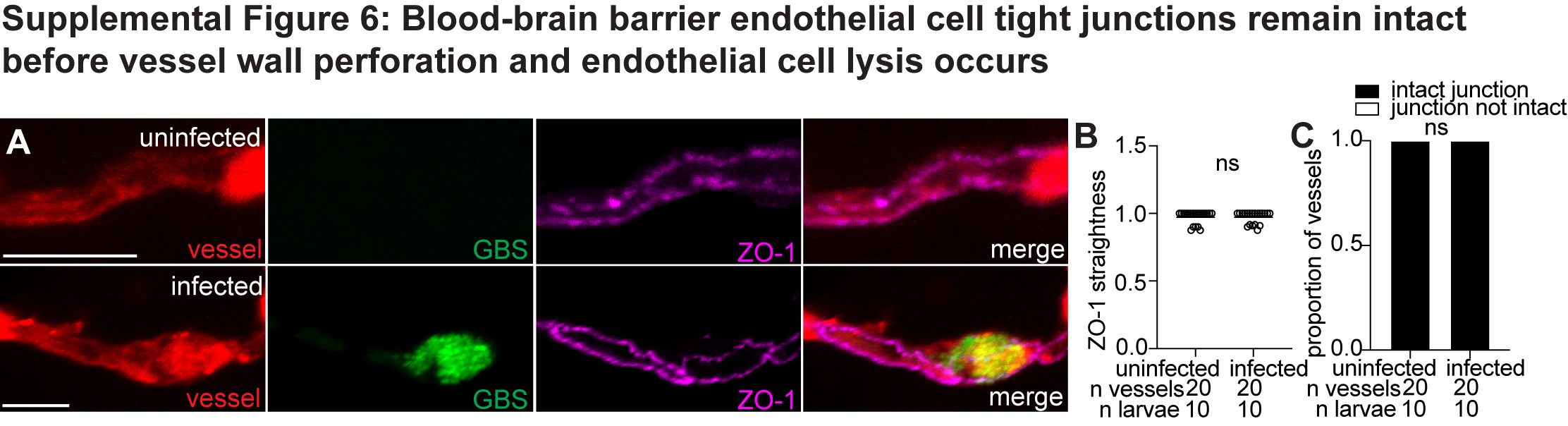

Supplement: S6 Fig — Blood–brain barrier endothelial cell tight junctions remain intact before vessel wall perforation and endothelial lysis occurs. (A) Representative confocal images of an uninfected (top) and infected (bottom) red fluorescent brain blood vessel from 14 hpi larvae infected with approximately 100 CFU GBS-GFP and fixed and stained with a ZO-1 Alexa647 antibody (pseudocolored magenta). Scale bars, 10 µm. (B) ZO-1 straightness in uninfected and GBS-infected vessels at 14 hpi. Horizontal bars, means; ns: not significant, Paired t test. (C) Proportion of uninfected or GBS-infected vessels with intact vessel tight junctions at 14 hpi; ns: not significant, Fisher’s exact test. All underlying data in S6 Fig can be found in the supplemental Excel file entitled “S1 Data”. (TIF) [file pbio.3003236.s006.tif]

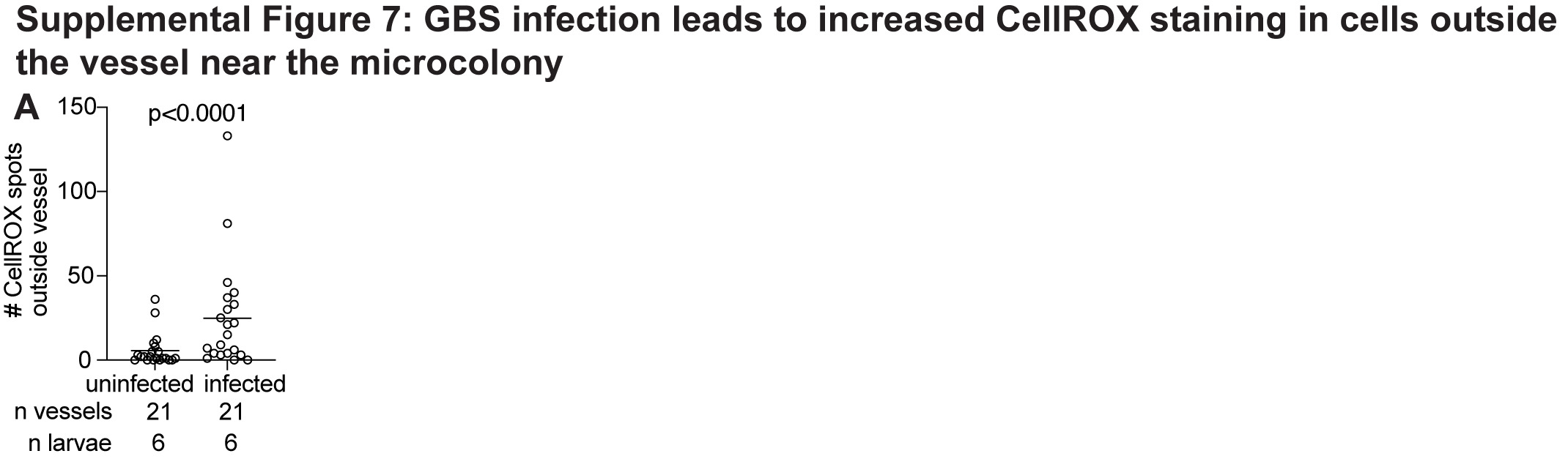

Supplement: S7 Fig — (A) Quantification of CellROX positive staining in non-endothelial cells around the microcolony. Horizontal bars, means; Student t test. All underlying data in S7 Fig can be found in the supplemental Excel file entitled “S1 Data”. (TIF) [file pbio.3003236.s007.tif]

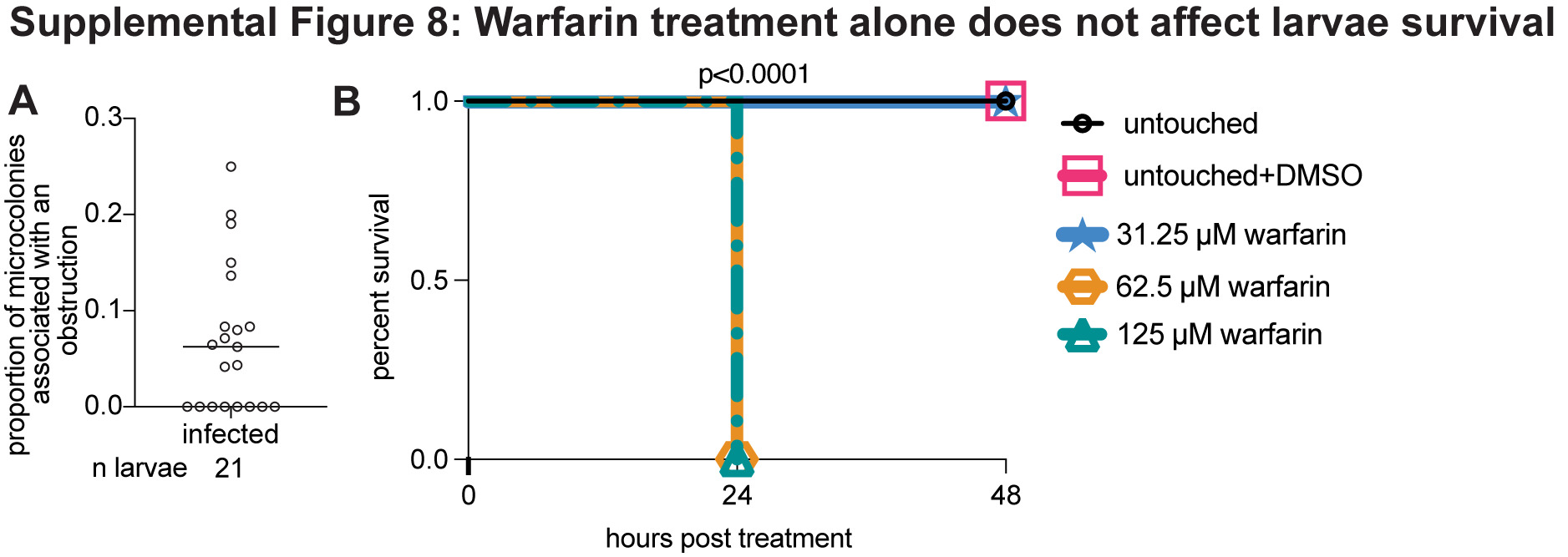

Supplement: S8 Fig — (A) Proportion of GBS microcolonies associated with an obstruction. Horizontal bar, mean. Representative of 2 independent experiments. (B) 48-h survival curve of larvae infected with approximately 100 CFU GBS and left untouched or treated (via soaking) with fish water containing 0.02 M DMSO and 31.25 µM warfarin, 62.5 µM warfarin, or 125 µM warfarin. n = 24 larvae per group; Kaplan–Meier test, compared to untouched group. All underlying data in S8 Fig can be found in the supplemental Excel file entitled “S1 Data”. (TIF) [file pbio.3003236.s008.tif]
